# Clostridium difficile in patients with nosocomial diarrhea, Northwest of Iran

**DOI:** 10.34172/hpp.2020.24

**Published:** 2020-03-30

**Authors:** Yalda Hematyar, Tahereh Pirzadeh, Seyyed Reza Moaddab, Mohammad Ahangarzadeh Rezaee, Mohammad Yousef Memar, Hossein Samadi Kafil

**Affiliations:** ^1^Student Research Committee, Tabriz University of Medical Sciences, Tabriz, Iran; ^2^Immunology Research Center, Tabriz University of Medical Sciences, Tabriz, Iran; ^3^Biotechnology Research Center, Faculty of Paramedicine, Tabriz University of Medical Sciences, Tabriz, Iran; ^4^Drug Applied Research Center, Faculty of Medicine, Tabriz University of Medical Sciences, Tabriz, Iran

**Keywords:** Clostridium difficile, Incidence, Immuno-chromatographic test, Toxin A, Toxin B protein, Diarrhea

## Abstract

**Background:** Clostridium difficile is known as a prevalent pathogen leading to infections ranging from mild diarrhea to severe disease and death. The aim of the present study was to evaluate the incidence of C. difficile from inpatients with nosocomial diarrhea hospitalized in different wards in the northwest region of Iran.

**Methods:** In this cross-sectional study, 485 diarrheal stool samples were collected from 384 patients referred from different wards of Imam Reza, Sina and Pediatric hospitals, Tabriz and transferred to the laboratory from 25 March 2015 till 1 March 2018. Immuno-chromatographicassay for detection of toxins A and B of C. difficile was used for identification.

**Results:** Clostridium difficile was isolated from 24 (4.7%) out of 485 samples. Fifteen patients(62.5%) were males and 9 were females (37.5%). Twelve positive patients were from the gastrointestinal ward (50%), 5 patients (20.8%) from surgery ward, 3 patients from infectious disease ward (12.5%), 3 patients from rheumatology ward (12.5%) and 1 patient (4.1%) were collected from neurology ward. 95.3% of diarrhea samples had no signs from toxin A and B.

**Conclusion:** These results indicate most of infected patients were from the gastrointestinaland surgery wards which show a different pattern of infection compared to previous studies.The neurology department had the lowest rate of infection. C. difficile is a health threat afterantibiotic consumption and for health promotion, developing strategies for less antibioticconsumption and preventing these emerging infections is critical. The low rate of this infection shows improvement in knowledge and effect of stewardships in physicians.

## Introduction

*Clostridium difficile* is an anaerobic gram-positive spore-forming rod that is part of normal flora in the human intestine in 3% of healthy individuals, 15%-20% of infants and in 10% to 30% chronically ill or hospitalized people. When broad spectrum antibiotics are used to treat infection, they suppress the normal flora of intestine along with infective organisms.^1,2^ Therefore, it can be considered as population treating criteria due to increase in antibiotic consumption and public access of antibiotics. The spectrum of *C. difficile* -associated disease (CDAD) ranges different diseases from pseudomembranous colitis, toxic megacolon, severe abdominal pain, bowel perforation or asymptomatic infection to diarrhea (the most frequent clinical symptom), and death.^3^ Many risk factors like antibiotic exposure including clindamycin, cephalosporins, and fluoroquinolones, advanced age, duration of hospitalization, severe underlying disease, chemotherapy, gastrointestinal surgery, Inflammatory bowel disease, Immunosuppression and gastric acid suppression are associated with CDAD.^4-6^ Toxins A and toxin B are the main virulence determinants in CDAD encoded by the *tcdA* and *tcdB* genes respectively. Toxin A is a type of enterotoxins, and toxin B is categorized as both of cyto- and enterotoxins.^7^ It is assumed that these toxins can cause colonic mucosal injury in colon and inflammation. *C. difficile* without these toxins has no pathogenicity.^8^ Mechanism of action of these toxins are as glucosyltransferase which can catalyzes the monoglucosylation of threonine 35/37of small GTP-binding proteins Rho, Rac, and Cdc42 within target cells, resulting cell death by modulation of different physiological cellular events.^9^ Toxin B has effect on monocyte by activation of cytotoxin release and destruction of the cytoskeleton even more than enterotoxin A, and consequently causes tissue injury in colon.^10^ Binary toxin is another toxin encoded by *ctdA* and *ctdB* . Its role in the pathogenesis is unclear; however, presence of this toxin among BI/NAP1/027 epidemic strains indicated its possible synergism with toxins A and B in cases of severe colitis.^11^ Number of *C. difficile* antibiotic-associated diarrhea cases are increasing in health care settings during past years.^12^ Different laboratory techniques may be used to diagnose fecal toxins A (TcdA) and B (TcdB). Gold standard method for biological detection of the *C. difficile* in the CDAD cases is cell cytotoxicity assay; however, this method is expensive and time consuming (takes 1-3 days).^13^ Stool culture is another procedure to detect toxigenic *C. difficile* in laboratories, by culture in cycloserine-cefoxitin- fructose agar. This method has high sensitivity, but it has low specificity, because of the false positive cases due to asymptomatic carriage of *C. difficile* , especially in hospitalized patients.^14^ Immuno-chromatography test is a recent advance in identification of this pathogen such as ImmunoCard Toxins A&B. This method can easily detect TcdA and TcdB in stool samples in about 20 minutes, which is fast and trustable method for identification. Early diagnosis of CDAD is really necessary in the hospital, since it can result on early treatment of this nosocomial infections and control of possible transmission.^7,15^ Due to the lack of knowledge on rate of this infection in the region and factors related to its establishment, in the present study, we aimed to investigate the presence and incidence of *C. difficile* in different wards as consequence of antibiotic-associated diarrhea by using immuno-chromatographic assay in the northwest of Iran.

## Materials and Methods

This cross-sectional study was conducted at Imam Reza hospital 800-bed university-affiliated, in Tabriz from 25 March 2015 to 1 March 2018. Also, samples from Sina hospital and Pediatric hospital were carried to the center for further investigation. The sampling technique was consecutive non-probability. A total of 485 stool specimens from 384 patients were included in the study. The toxin A and B were detected by immuno-chromatographic test IMMUNOQUICK^®^ TOX A & B (BioSynex, Strasbourg, France). The procedure of *C. difficile* toxin detection was conducted according to the company protocol along with positive and negative controls. The device which was used in this study had immobilized antibodies in a reaction port with two stripes. Test stripe had antibodies against *C. difficile* toxins A and B and a control stripe with anti-IgG antibodies. Antibioses against toxins A and B coupled to horseradish peroxidase were the conjugate. Data were entered and analyzed in IBM SPSS^®^ 23.0 (Statistical Package for the Social Sciences,SPSS Inc., Chicago, Ill., USA). The quantitative variable of age was presented as mean + standard deviation (SD), whereas gender and presence of *C. difficile* were presented as frequency and percentages. Data were stratified for age, gender and ward. Odd ratio (OR) & 95% confidence interval (CI) were determined to assess the associated risk. Chi-square test was applied to determine significant age, gender groups and ward.

## Results

**Figure 1:**
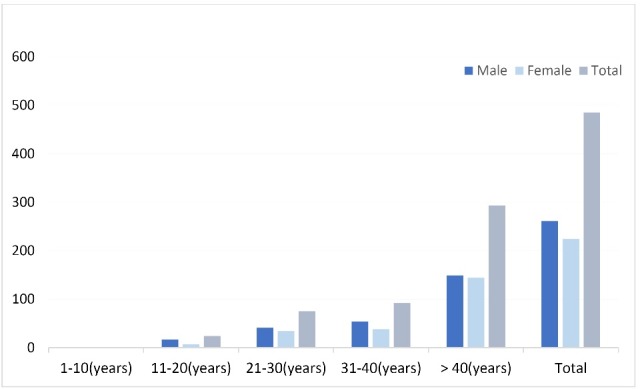
Frequency of investigated stool samples for *C. difficile* on the basis of sex and age.

Among the 485 samples collected, 261 (53.7%) were male and 224 (46.3%) were female and the mean age was 43 (SD 3) years old. Overall, 98.3% of the patients were hospitalized (Table 1 and Figure 1). In this study, toxin production of *C. difficile* was detected by the immuno-chromatographic test in 24 patients (4.9%), all of which included hospitalized patients. Of these, 15 patients (62.5%) were males and 9 were females (37.5%). Twelve patients in the gastrointestinal ward (50%), 5 patients (20.8%) in the surgery, 3 patients in the infectious (12.5%), 3 patients in the rheumatology (12.5%) and 1 patient (4.2%) was admitted to the neurology department. No significant differences were observed in the presence of toxin with sex, outpatient or hospitalization and hospital admission (Table 1). 95.8% of infected patients had a history of antibiotic consumption including cephalosporins, ciprofloxacin, and co-amoxiclav.

## Discussion

*Clostridium difficile* is a common bacterium which can be found in most of people’s colon without any symptoms, but can cause severe diarrhea and colitis in some persons. This organism is usually acquired as a nosocomial infection in the hospital. In most of cases environment contamination is the source of infection by health care workers which carry this pathogen in their hands, or on contaminated instruments. The most common methods for the clinical diagnosis of *C. difficile* are immuno-chromatography, ELISAs, immunoenzymic, and cytotoxicity (cell culture) assay methods. These methods are popular because of their rapid, cost-effective, and easy performance properties.^16^ *C. difficile* infection, especially CDAD has different prevalence worldwide. In our study, *C. difficile* infection was detected in 4.9% of the patients with diarrhea. A previous study in Iran regarding presence of *C .difficile* , 6.1% of patients were positive with nosocomial diarrhea and 4% of children with diarrhea had this bacterium.^17,18^ In a report from Turkey, 4.9% for patients with diarrhea were positive which was consistent with our results.^19^ In another study by Gursoy et al, 27.7% of the patients were positive for *C. difficile* .^20^ In a study among Brazilian patients, *C. difficile* infection was positive in 5.5% of hospitalized children with acute diarrhea. In Argentina, 38.5% of symptomatic patients were positive for *C. difficile* .^3,21^

These differences were due to the limitations and methods of studies. We used an immuno-chromatography test for screening*C. difficile* toxins A and B. Regardless of initial negative results in immuno-chromatography, additional toxin-positive cases could be revealed. Some studies use methods with high sensitivity for bacterial detection, but the presence of bacteria is not equal to disease and toxin production is a critical point. Moreover, strains obviously produced fewer toxins even after toxigenic culture, resulting in negative immuno-chromatography test results.^22^

In the present study, positive cases were from gastrointestinal, general surgery, infectious, rheumatology and neurology wards. Use of antimicrobial agents in patients with ≥2 days diarrhea is a marker of CDAD. Cephalosporin’s, ciprofloxacin and co-amoxiclav were the main responsible antibiotic of CDAD. In 95.8% of cases, we had a history of antibiotic consumption and only one case had no history of antibiotic treatment. Though treatment is successful in most of CDAD, but *C. difficile* colitis can be considered as serious treat for health, especially when diagnose and treatment of the patients postponed.^3^ *C. difficile* is an important intestinal pathogen in patients in our hospitals. Toxin production in *C. difficile* strains are determined as the major cause of severe diarrhea, and antibiotic consumption gives this bacterium an opportunity of growth. Therefore, the best administration approach is to limit the use of broad-spectrum antibiotics, public education for physicians and stewardships and guidelines for antibiotic consumption in hospitals. Growing number of *C. difficile* infection worldwide, emphasize importance of further studies on its epidemiological and pathological properties of infection. These results indicate most of the infected patients were from gastrointestinal and surgery wards which show different patterns of infection compared to previous studies. The neurology department had the lowest rate of infection. *C. difficile* is a health threat after antibiotic consumption and for health promotion, developing strategies for less antibiotic consumption and preventing these emerging infections is critical. However, the low rate of this infection shows improvement in the knowledge and effect of stewardships in control of infection. Restricted access to the antibiotics with risk of CDAD is critical to control this infection. In conclusion, it is necessary to develop our knowledge on the mechanisms involved in pathogenesis of *C. difficile* . Still too many gaps are present on role of toxins and mechanism of pathogenesis by this bacterium. Therefore, it will be necessary to evaluate the epidemiology and to implement measures to control the nosocomial spread of this infection. The main limitation of this study was inability to collect outpatient’s sample and study how many cases of CDAD in population is present. Indeed, further studies on rate of CDAD among population are critical and will help us to develop local policies to control this infection. Results of the present study shows CDAD as risk factor for patients hospitalized in our gastrointestinal and surgery wards and shows importance of policies regarding antibiotic stewardship in these wards to prevent this health treating infection.

## Ethical approval

This study was approved by the Ethic Committee of Tabriz University of Medical Sciences with reference number IR.TBZMED.REC.1397.453.

## Competing interests

None to declare.

## Funding

This study was supported by Drug Applied Research Center, Tabriz University of Medical Sciences with reference number 60429.

## Authors’ contributions

YH: Data collection, sample preparation, analysis, Manuscript preparation, Final proof of the manuscript. TP: supervision, Funding, Final proof of the manuscript. SRM: Manuscript preparation, Final proof of the manuscript. MAR: supervision, Final proof of the manuscript. MYM: sample preparation, analysis, Manuscript preparation, Final proof of the manuscript. HSK: Concept, supervision, Manuscript preparation, Final proof of the manuscript.

## Acknowledgments

We thank all collaborations by staff of Imam Reza hospital and Drug Applied Research Center.

**Table 1:** Gender of patients with *Clostridium difficile* associated diarrhea in this study

**Sex**	**Result**					
	**Positive**		**Negative**		**Total**	
	**No.**	**%**	**No.**	**%**	**No.**	**%**
Male	15	62.5	246	53.36	261	53.7
Female	9	37.5	215	46.63	224	46.3
Total	24	4.9	461	95.1	485	100
